# Relative effectiveness of bivalent boosters against severe COVID-19 outcomes among people aged ≥ 65 years in Finland, September 2022 to August 2023

**DOI:** 10.2807/1560-7917.ES.2024.29.37.2300587

**Published:** 2024-09-12

**Authors:** Eero Poukka, Jori Perälä, Hanna Nohynek, Sirkka Goebeler, Kari Auranen, Tuija Leino, Ulrike Baum

**Affiliations:** 1 Infectious Disease Control and Vaccinations Unit, Department of Health Security, Finnish Institute for Health and Welfare, Helsinki, Finland; 2 Department of Public Health, Faculty of Medicine, University of Helsinki, Helsinki, Finland; 3 Forensic Medicine Unit, Department of Government services, Finnish Institute for Health and Welfare, Helsinki, Finland; 4 Department of Clinical Medicine, University of Turku, Turku, Finland; 5 Department of Mathematics and Statistics, University of Turku, Turku, Finland

**Keywords:** COVID-19, vaccine effectiveness, bivalent, hospitalisation, death, Finland, coronavirus disease (COVID-19), vaccines and immunisation, epidemiology

## Abstract

**Background:**

Long-term effectiveness data on bivalent COVID-19 boosters are limited.

**Aim:**

We evaluated the long-term protection of bivalent boosters against severe COVID-19 among ≥ 65-year-olds in Finland.

**Methods:**

In this register-based cohort analysis, we compared the risk of three severe COVID-19 outcomes among ≥ 65-year-olds who received a bivalent booster (Original/Omicron BA.1 or Original/BA.4–5; exposed group) between 1/9/2022 and 31/8/2023 to those who did not (unexposed). We included individuals vaccinated with at least two monovalent COVID-19 vaccine doses before 1/9/2022 and ≥ 3 months ago. The analysis was divided into two periods: 1/9/2022–28/2/2023 (BA.5 and BQ.1.X predominating) and 1/3/2023–31/8/2023 (XBB predominating). The hazards for the outcomes between exposed and unexposed individuals were compared with Cox regression.

**Results:**

We included 1,191,871 individuals. From 1/9/2022 to 28/2/2023, bivalent boosters were associated with a reduced risk of hospitalisation due to COVID-19 (hazard ratio (HR): 0.45; 95% confidence interval (CI): 0.37–0.55), death due to COVID-19 (HR: 0.49; 95% CI: 0.38–0.62), and death in which COVID-19 was a contributing factor (HR: 0.40; 95% CI: 0.31–0.51) during 14–60 days since vaccination. From 1/3/2023 to 31/8/2023, bivalent boosters were associated with lower risks of all three severe COVID-19 outcomes during 61–120 days since a bivalent booster (e.g. HR: 0.53; 95% CI: 0.39–0.71 for hospitalisation due to COVID-19); thereafter no notable risk reduction was observed. No difference was found between Original/Omicron BA.1 and Original/BA.4–5 boosters.

**Conclusion:**

Bivalent boosters initially reduced the risk of severe COVID-19 outcomes by ca 50% among ≥ 65-year-olds, but protection waned over time. These findings help guide vaccine development and vaccination programmes.

**Key public health message**

**What did you want to address in this study and why?**

To help develop COVID-19 vaccination programmes, we evaluated the long-term protection of bivalent boosters against severe COVID-19 outcomes among ≥ 65-year-olds. Those who received a bivalent booster were compared with those vaccinated with at least two monovalent COVID-19 vaccine doses before 1 September 2022. The analysis concerned two periods (September 2022–February 2023 and March–August 2023), when different variants of SARS-CoV-2 were circulating.

**What have we learnt from this study?**

The bivalent booster was initially associated with a ca 50% reduction in risk of hospitalisation due to COVID-19, death due to COVID-19 and death in which COVID-19 was a contributing factor among ≥ 65-year-olds, but the relative protection began to wane after 120 days since the bivalent booster. During March–August 2023, a bivalent booster given in autumn 2022 offered at most only minor additional protection against severe COVID-19 outcomes.

**What are the implications of your findings for public health?**

These findings help policymakers to develop COVID-19 vaccination programmes. They can be used for evaluation of both tailored COVID-19 vaccines and the need and timing for additional booster doses in the future.

## Introduction

Due to the emergence of new severe acute respiratory syndrome coronavirus 2 (SARS-CoV-2) variants with immune evasive capabilities [1], new, bivalent COVID-19 vaccines, containing mRNAs that encode the spike proteins of both the original virus strain and the Omicron variant, were developed in 2022. The European Medicines Agency authorised Original/Omicron BA.1 and Original/Omicron BA.4–5 bivalent vaccines in September 2022 [2], and these were promptly recommended in Finland for all people aged ≥ 65 years who had neither received a COVID-19 vaccine dose nor been infected with SARS-CoV-2 during the previous 3 months.

In previous studies, bivalent boosters have been found to increase protection against severe COVID-19 outcomes [3-10]. However, the duration of protection has remained unclear, and the protection is expected to vary by time since vaccination and across different Omicron lineages [10-12]. Therefore, studies estimating the effectiveness of bivalent boosters over time are needed as policymakers are considering recommendations for COVID-19 vaccinations in future seasons. During 2022/23, COVID-19 vaccination policies varied between countries: some countries, such as Finland, recommended only one bivalent booster dose in autumn 2022 [13], while few recommended a second booster in spring 2023 [14].

The aim of this study was to estimate the relative effectiveness of a bivalent booster against severe COVID-19 outcomes, namely hospitalisation due to COVID-19, death due to COVID-19 and death in which COVID-19 was a contributing factor, among people aged ≥ 65 years in Finland, based on national register data.

## Methods

### Study design

We conducted a population-based cohort analysis linking the national register data from Finland, which uses people’s unique person identifier. The study period was from 1 September 2022 to 31 August 2023. In analogy to our previous studies [15,16], we assembled a cohort of individuals aged ≥ 65 years. We included only individuals who had received at least two monovalent COVID-19 vaccine doses before 1 September 2022. These vaccines were either Comirnaty (tozinameran, BNT162b2 mRNA, BioNTech-Pfizer, Mainz, Germany/New York, United States (US)), Spikevax (elasomeran, mRNA-1273, Moderna, Cambridge, US), or Vaxzevria (AZD1222, ChAdOx1 nCoV-19, Oxford-AstraZeneca, Cambridge, United Kingdom). In addition, we excluded individuals who were hospitalised due to COVID-19 at the beginning of the study or had received a COVID-19 vaccination with too short dosing interval (i.e. less than 91 days between two doses or less than 14 days between the first two doses for all vaccines, except if the first vaccination was with Vaxzevria, in this case this was less than 42 days) or a bivalent vaccination before the study, as described in Supplementary Table S1.

### Exposure

The exposure was defined as a vaccination with an Original/Omicron BA.1 or Original/Omicron BA.4–5 bivalent COVID-19 vaccine recorded in the Finnish Vaccination Register. The bivalent booster in this study was either Comirnaty or Spikevax as other bivalent vaccines were not available in Finland during the study period. The exposure was time-dependently categorised into 10 intervals: not vaccinated with a bivalent booster (the reference), and 0–2, 3–7, 8–13, 14–60, 61–120, 121–180, 181–240, 241–300, and 301–364 days since bivalent booster. The three short intervals in the first 2 weeks since the bivalent booster were negative control exposures and used to investigate the possibility of time-varying confounding as observed in previous studies [9,11,17,18]. In these studies, a reduced risk of severe COVID-19 outcomes has been observed immediately after vaccination before any plausible immune response [19], indicating time-varying confounding.

### Outcomes of interest and negative control outcome

The severe COVID-19 outcomes (outcomes of interest) included hospitalisation due to COVID-19, death due to COVID-19, and death in which COVID-19 was a contributing factor.

Hospitalisations, recorded in the Care Register for Health Care, had to fulfil the following two criteria to be considered as hospitalisations due to COVID-19: (i) the primary diagnosis was COVID-19 (International Classification of Diseases, 10th revision (ICD-10) codes: U07.1, U07.2), acute respiratory tract infection (ICD-10 codes: J00–J22, J46) or severe complication of lower respiratory tract infections (ICD-10 codes: J80–84, J85.1, J86) and (ii) a positive PCR- or antigen SARS-CoV-2 sample was obtained from the hospitalised patient in the period extending from 14 days before to 7 days after the hospital admission and registered in the National Infectious Diseases Register (Table 1).

**Table 1:** Registers used for outcome and covariate definition, when investigating relative effectiveness of bivalent boosters against severe COVID-19, Finland, 1 September 2022−31 August 2023

Characteristics	Data source	Data update
Outcomes		
Hospitalisation due to COVID-19	National Infectious Diseases Register	End of study (31 August 2023)
	Care Register for Health Care	End of study
Death due to COVID-19	Death certificates	End of study
Death in which COVID-19 was a contributing factor	Death certificates	End of study
Emergency room visit due to injury	Care Register for Health Care	End of study
Covariates		
Age	Population Information System	Baseline (31 August 2022)
Region of residency	Population Information System	Baseline
Sex^a^	Population Information System	Baseline
Hospitalisation between 1 September 2020 and 31 August 2022	Care Register for Health Care	Baseline
- Presence of severely immunocompromising conditions^b^ - Number of highly predisposing medical conditions for severe COVID-19^c^ - Number of moderately predisposing medical conditions for severe COVID-19^d^	Care Register for Health Care	Baseline
	Care Register of Primary Health Care visits	Baseline
	Special Reimbursement Register for Medicine Expenses	30 November 2020
	Prescription Centre database	30 November 2020
Residency in a long-term care facility	Care Register for Social Care	31 December 2021
Seasonal influenza vaccination in 2022/23	Finnish Vaccination Register	End of study (31 August 2022)
Number of monovalent COVID-19 vaccinations	Finnish Vaccination Register	Baseline
Last laboratory-confirmed SARS-CoV-2 infection before the study	National Infectious Diseases Register	Baseline

SARS-CoV-2: severe acute respiratory syndrome coronavirus-2.

^a^ Sex was collected as binary (male/female).

^b^ These conditions are listed in Supplementary Table S2.

^c^ These conditions are listed in Supplementary Table S3.

^d^ These conditions are listed in Supplementary Table S4.

To define the two COVID-19 death outcomes, we exclusively used data collected from death certificates, which provide the most precise information related to COVID-19 deaths. In Finland, physicians record the cause of death of their patients as well as other significant conditions contributing to death in death certificates that are subsequently reviewed by medico-legal specialists at the Finnish Institute for Health and Welfare. In our study, death due to COVID-19 included all deaths in which COVID-19 (ICD-10 codes: U07.1, U07.2, U09, and U10) was recorded as the immediate or underlying cause of death in the death certificate. Cases for whom COVID-19 was a contributing factor to death (i.e. the cause of death was another reason than COVID-19, but COVID-19 had contributed to the death) were equally retrieved from the death certificates. The data from the death certificates were stored into a database by medico-legal specialists.

In addition, we defined a fourth endpoint, which we assumed to be unaffected by the exposure. This negative control outcome was any emergency room visit due to injury (ICD-10 codes: S00–T14) recorded in the Care Register for Health Care.

### Covariates

We considered the following covariates as confounders (Table 1): age (continuous variable, in years), region of residency, sex (male/female), hospitalisation between 1 September 2020 and 31 August 2022, presence of severely immunocompromising conditions – as detailed in Supplementary Table S2, number of highly predisposing medical conditions for severe COVID-19 – as specified in Supplementary Table S3, number of moderately predisposing medical conditions for severe COVID-19 – as presented in Supplementary Table S4, residency in a long-term care facility, seasonal influenza vaccination in 2022/23, number of monovalent COVID-19 vaccinations, and the last laboratory-confirmed SARS-CoV-2 infection before the study categorised into no infection, pre-Omicron (before 2022), and Omicron infection (since 2022).

### Individual follow-up

The individual follow-up period started earliest on 1 September 2022 and at least 90 days after either (i) the last monovalent COVID-19 vaccination or (ii) laboratory-confirmed SARS-CoV-2 infection, both having occurred before the study. Each individual was followed until:

• outcome of interest;

• day 14 after laboratory-confirmed SARS-CoV-2 infection if the outcome of interest was hospitalisation due to COVID-19;

• day 60 after laboratory-confirmed SARS-CoV-2 infection for the other outcomes of interest (i.e. death due to COVID-19 or death where COVID-19 was a contributing factor);

• a second bivalent booster;

• a monovalent vaccination;

• death, or 

• 31 August 2023, whichever occurred first.

### Statistical analysis

We compared the hazard of the three severe COVID-19 outcomes between exposed (bivalent vaccinated) and unexposed individuals allowing different hazard ratios (HR) for each of the nine intervals since bivalent booster. In addition, the analysis was done separately for the two calendar time periods, September 2022–February 2023 (mostly predominated by BA.5 and BQ.1.X, as illustrated in Supplementary Figure S1) and March–August 2023 (predominated by XBB). The HR was estimated using Cox regression with calendar time as the underlying time scale and adjusted for the aforementioned covariates. Additionally, we stratified by age differentiating between individuals aged 65–79 years and those aged ≥ 80 years and analysed the HR separately for the Original/BA.1 and Original/BA.4–5 bivalent vaccines. For these additional analyses, we merged the first three time-since-vaccination intervals from 0 to 13 days since the bivalent booster (for negative control exposures).

To evaluate the presence of residual confounding, we estimated the HR for the negative control outcome and expected to find no difference between the unexposed and exposed. The analysis was conducted as described above considering the negative control outcome as the outcome of interest. All analyses were performed in R 4.3.2 (R Foundation for Statistical Computing, Vienna, Austria).

## Results

The study cohort included 1,191,871 people aged 65–110 years of whom 4% were living in a long-term care facility and 8% had severely immunocompromising conditions (Table 2). Only a small proportion (n = 81,764; 7%) had received a laboratory-confirmed SARS-CoV-2-positive test result before the study. In the study cohort, the 2022/23 influenza vaccination coverage reached 59% (n = 704,101) during the study period.

**Table 2:** Distribution of baseline characteristics of the study cohort, i.e. people aged ≥ 65 years living in Finland during September 2022–August 2023 (n = 1,191,871)

Characteristic	Number	Proportion (%)
**Age in years**		
65–79	886,240	74
80–110	305,631	26
**Region of residence**		
Helsinki-Uusimaa	285,668	24
Åland	6,655	1
Northern and Eastern Finland	304,279	26
Southern Finland	281,241	24
Western Finland	314,028	26
**Sex**		
Female	663,141	56
Male	528,730	44
**Hospitalised between September 2020 to August 2022**		
No	923,953	78
Yes	267,918	22
**Presence of severely immunocompromising conditions^a^**		
No	1,090,769	92
Yes	101,102	8
**Number of highly predisposing medical conditions^b^**		
0	927,126	78
1	178,731	15
2–3	86,014	7
**Number of moderately predisposing medical conditions^c^**		
0	650,124	55
1	323,423	27
2	127,813	11
3–8	90,511	8
**Long-term care**		
No	1,139,655	96
Yes	52,216	4
**Number of monovalent COVID-19 vaccinations before the study**		
2	64,932	5
3	467,769	39
4–5	659,170	55
**Last laboratory-confirmed SARS-CoV-2 infection before the study**		
No prior infection	1,110,107	93
Pre-Omicron infection	13,453	1
Omicron infection	68,311	6

SARS-CoV-2: severe acute respiratory syndrome coronavirus 2.

^a^ Severely immunocompromising conditions are described in Supplementary Table S2.

^b^ Highly predisposing medical conditions are described in Supplementary Table S3.

^c^ Moderately predisposing medical conditions are described in Supplementary Table S4.

During the study, 652,746 (55%) of the study subjects received a bivalent booster. Approximately one third (n = 194,383) received Comirnaty BA.1 while two thirds (n = 455,665) received Comirnaty BA.4–5. Spikevax bivalent boosters were used only in small numbers (n = 2,698 < 1%). The median time since the bivalent booster until the end of follow-up was 284 days (interquartile range (IQR): 267–297 days). There were 3,137 hospitalisations due to COVID-19, 1,624 deaths due to COVID-19, and 1,307 deaths in which COVID-19 was a contributing factor.

**Figure 1:**
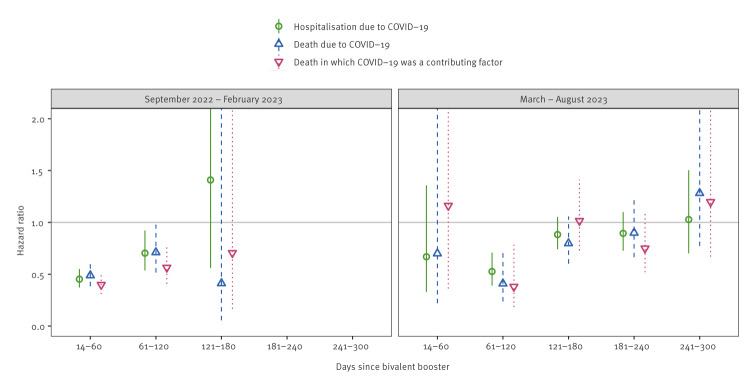
Hazard ratios (with 95% confidence intervals) for bivalent boosters against severe COVID-19 outcomes among people aged ≥ 65 years living in Finland during September 2022–February 2023 and March–August 2023 (n = 1,191,871)

In September 2022–February 2023, the bivalent booster was associated with a reduced rate of hospitalisation due to COVID-19 (HR: 0.45; 95% confidence interval (CI): 0.37–0.55), death due to COVID-19 (HR: 0.49; 95% CI: 0.38–0.62), and death in which COVID-19 was a contributing factor (HR: 0.40; 95% CI: 0.31–0.51) during 14–60 days since bivalent booster as shown in Supplementary Table S5 (Figure 1). For all severe COVID-19 outcomes, HRs were larger during 61–120 days since bivalent booster compared with the 14–60 days interval (HR: 0.70; 95% CI: 0.54–0.92; HR: 0.71; 95% CI: 0.52–0.98; HR: 0.56; 95% CI: 0.41–0.78, respectively). During 121–180 days since bivalent booster, the 95% CI were very wide and encompassed the value 1.0.

The hazard ratios for < 14 days since bivalent booster (no immune response and selection bias) and > 300 days since bivalent booster (exceeding the interquartile range of the maximum time since bivalent booster) are presented in Supplementary Table S5.

In March–August 2023, only a few severe COVID-19 outcomes were observed ≤ 60 days since bivalent booster, because most of the exposed people had received the bivalent booster in autumn 2022 and thus more than 60 days ago. The bivalent booster was associated with lower risks of hospitalisation due to COVID-19 (HR: 0.53; 95% CI: 0.39–0.71), death due to COVID-19 (0.41; 95% CI: 0.24–0.70), and death in which COVID-19 was a contributing factor (0.38; 95% CI: 0.18–0.78) during 61–120 days since bivalent booster (Figure 1, Supplementary Table S5), but the protective effect was not observed in later time intervals.

**Figure 2:**
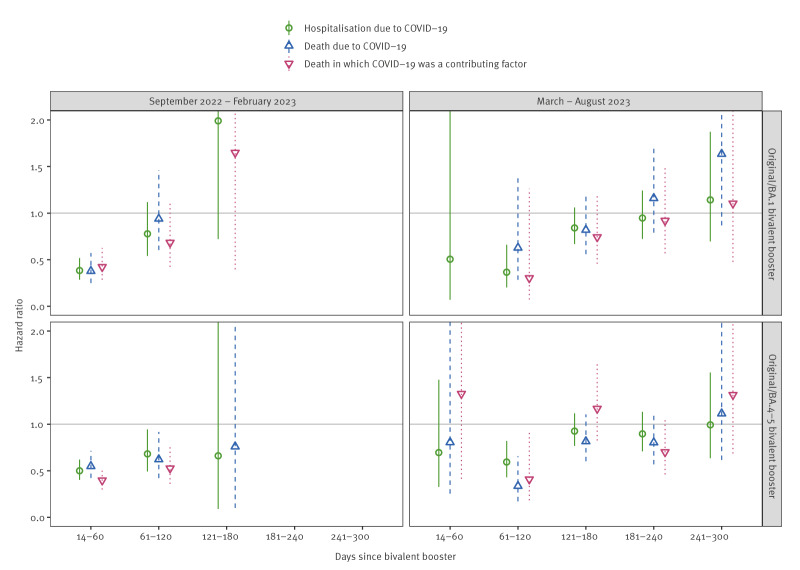
Hazard ratios (with 95% confidence intervals) for bivalent boosters against severe COVID-19 outcomes stratified by vaccine composition among people aged ≥ 65 years living in Finland during September 2022–February 2023 and March–August 2023 (n = 1,191,871)

**Figure 3:**
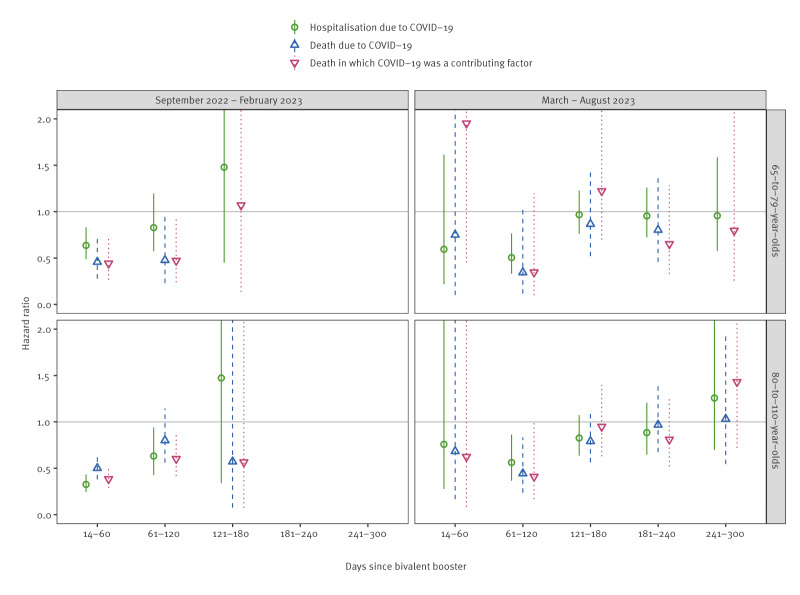
Hazard ratios (with 95% confidence intervals) for bivalent boosters against severe COVID-19 outcomes stratified by age group among people aged ≥ 65 years living in Finland during September 2022–February 2023 and March–August 2023 (n = 1,191,871)

Both the BA.1 and BA.4–5 bivalent vaccine boosters reduced the risk of severe COVID-19 outcomes equally; for example, the HRs for hospitalisation due to COVID-19 were 0.38 (95% CI: 0.28–0.52) for the BA.1 vaccine and 0.50 (95% CI: 0.40–0.62) for the BA.4–5 vaccine during 14–60 days since bivalent booster in September 2022–February 2023 as presented in Supplementary Table S6 (Figure 2). When stratified by age, we did not observe any major difference in the HRs for those aged ≥ 80 years and for those aged 65–79 years in either study period as shown in Supplementary Table S7–S8 (Figure 3).

The hazard ratios for < 14 days since bivalent booster (no immune response and selection bias) and > 300 days since bivalent booster (exceeding the interquartile range of the maximum time since bivalent booster) are presented in Supplementary Table S6.

The hazard ratios for < 14 days since bivalent booster (no immune response and selection bias) and > 300 days since bivalent booster (exceeding the interquartile range of the maximum time since bivalent booster) are presented in Supplementary Tables S7–S8.

In the negative control outcome analysis, we observed 30,399 emergency room visits due to injury. We found no major difference in the risk of injury ≥ 14 days since bivalent booster among people who received the booster when compared with those who did not; all HR point estimates were close to 1.0 (Supplementary Table S5).

In the negative control exposure analysis, the days 0–13 since bivalent booster were associated with statistically significantly reduced rates for all three severe COVID-19 outcomes (Supplementary Table S5). The HR ranged between 0.11 and 0.54 during September 2022–February 2023 with the risk being lowest during 0–2 days since bivalent booster. Similarly, the risk of injury was reduced during 0–2 days since bivalent booster (0.59; 95% CI: 0.48–0.72 in September 2022–February 2023, Supplementary Table S5). However, during 3–7 and 8–13 days since bivalent booster the corresponding HR was close to 1.0.

## Discussion

In our 12-month follow-up study of people ≥ 65 years who had received at least two monovalent COVID-19 vaccine doses, a bivalent booster was initially associated with a ca 50% reduction in the risk of severe COVID-19 outcomes, but this relative protective effect was attenuated after 120 days. Particularly during March–August 2023, when XBB was predominant, a bivalent booster given in autumn 2022 demonstrated no notable protective effect. The BA.1 and BA.4–5 bivalent vaccines seemed to provide generally comparable protection against severe COVID-19 outcomes. For individuals aged ≥ 80 years the bivalent booster seemed to be at least as protective as for those aged 65 to 79 years.

We suggest three hypotheses for the reduced protective effect of the bivalent booster among people ≥ 65 years after 120 days since bivalent booster. Firstly, the protection might have waned over time since bivalent booster as reported by previous studies [8,10,12,17]. Secondly, at the beginning of the study, BA.5 was the predominant Omicron lineage in Finland, but in 2023 other Omicron lineages (i.e. BQ.1, CH.1.1 and XBB) with higher immune evasive capabilities became more prevalent [20-23]. Because the potential effect of waning coincided with the emergence of the new lineages, we were not able to separate these effects from each other. A test-negative design study conducted between December 2022 and April 2023 in the United Kingdom (UK) was able to distinguish between the aetiological lineages and indicated that the relative vaccine effectiveness of Original/BA.1 bivalent booster against severe COVID-19 caused by BQ.1, CH.1.1 and XBB might be slightly lower than that against BA.5 [10]. However, in this study the estimates for BA.5 and other lineages could not be directly compared, illustrating the difficulty to separate the effects of different SARS-CoV-2 lineages on COVID-19 vaccine protection. Thirdly, the prevalence of hybrid immunity, i.e. the combination of protection obtained via both vaccination and SARS-CoV-2 infection, increased during the study period due to the high incidence of SARS-CoV-2 infections [24]. As many SARS-CoV-2 infections were not laboratory confirmed and thus not registered in the National Infectious Diseases Register [25], the effect of hybrid immunity could not be properly assessed in our study. It is, however, likely that hybrid immunity was more prevalent among the unexposed because individuals with a recent SARS-CoV-2 infection were less likely to seek the bivalent booster. Because hybrid immunity provides protection against severe COVID-19 outcomes [26], its increased prevalence among the unexposed likely attenuated the measured benefit of the bivalent booster by some margin.

Besides this study, only few studies have evaluated the bivalent vaccine effectiveness against severe COVID-19 outcomes after XBB became predominant. A study by Fabiani et al. [12] conducted between April and June 2023 reported a similarly low relative effectiveness of the bivalent booster after 120 days among people aged ≥ 60 years living in Italy (relative vaccine effectiveness: 17%; 95% CI: 8–25%, during 121–180 days since bivalent booster), with comparable effectiveness observed among those aged 60–79 years and those aged ≥ 80 years. Likewise, a corresponding study conducted among those aged ≥ 50 years in the UK estimated the relative effectiveness at 21% (95% CI: 10–31%) after 14 weeks [10], i.e. after ca 100 days. In another recent study from the UK conducted between April and August 2023, Kirsebom et al. [27] observed increased protection (relative effectiveness: 30–50%) after a second bivalent booster given in spring among individuals aged ≥ 75 years when compared with those who had received only the first bivalent booster in autumn. Given the potential waning after 120 days, enhancing the protection with an additional booster after 4–6 months might thus avert even more cases of severe COVID-19 among older adults. However, in making decisions about two annual vaccinations (i.e. autumn and spring boosters) policymakers should consider the epidemiological situation, increased workload for healthcare workers caused by the additional vaccinations, cost-effectiveness, and attenuation of the protective effect of the vaccine due to immune imprinting [28,29].

A major limitation in our study is that the applied covariate adjustment did not sufficiently control time-varying confounding. During 0–7 days since bivalent booster, i.e. before any plausible immune response [19], we observed a protective effect against severe COVID-19 outcomes, indicating the presence of confounding. Similar (time-varying) confounding effects have been previously observed in multiple other studies [9,11,17,18,27]. It is likely that these confounding effects were partly caused by selection bias as individuals with acute respiratory symptoms, a predeterminant of severe COVID-19 outcomes, were not advised to seek the bivalent booster [27]. However, as we detected confounding also in the negative control outcome analysis (in which the reduced risk of injury should not have been caused by a similar selection bias), additional time-varying confounders may exist. Another hypothesis is that temporal side effects due to the vaccination (e.g. fever) might have affected the behaviour of the recently vaccinated individuals (e.g. isolation) which could have led to decreased exposure to SARS-CoV-2 and decreased the risk of the negative control outcome (i.e. injury). As the understanding of this time-varying confounding bias is currently limited, methods for controlling it should be introduced in the future. To interpret our study, it should be noted that the effect of confounding likely diminishes over time, as observed in the negative control outcome analysis, and likely becomes negligible after 13 days since bivalent booster.

Another limitation is that only a small proportion of all SARS-CoV-2 infections were reported to the National Infectious Diseases Register during the study period [25], as laboratory testing for SARS-CoV-2 was not recommended for mild COVID-19 cases. Instead, lateral flow tests (i.e. home tests), whose results have not been notifiable, were widely used in Finland, also in long-term care, where the COVID-19-related mortality has been high. We therefore relied on death certificate data when defining study outcomes death due to COVID-19 and death in which COVID-19 was a contributing factor but also utilised the available register data to censor the individual follow-up after an infection to partly control for the effect of hybrid immunity. It should additionally be noted that the bivalent booster might have been slightly more accessible to vulnerable individuals (e.g. those living in long-term care), although this selection was unlikely substantial as there was no shortage of bivalent vaccines. Furthermore, some covariate data, such as residency in long-term care, were not updated at the baseline (Table 1) which increased risk of confounding in the analyses. Finally, the number of severe COVID-19 outcomes was relatively small during March–August 2023 as shown in Supplementary Figure S2, which led to unprecise estimates for that period.

Our study has multiple strengths. It was timely and representative, and we used the monovalent vaccinated as the reference group whose characteristics are probably more similar to the characteristics of the bivalent-vaccinated compared with those of the unvaccinated. We also included a negative control outcome analysis in the study and found no signs of major residual confounding after 13 days since bivalent booster. The recording of vaccinations and COVID-19 outcomes was mandatory, and the used registers have been well maintained as they have been used for routine surveillance of the COVID-19 vaccination programme and the COVID-19 pandemic in Finland [3,15,16].

## Conclusions

In 2022/23, a bivalent booster was initially associated with a ca 50% reduction in the risk of severe COVID-19 outcomes among older adults in Finland. However, this relative protective effect waned over time, and during March–August 2023, a bivalent booster given in autumn 2022 offered at most only minor additional protection against severe COVID-19 outcomes. These findings can support policymakers to develop COVID-19 vaccination strategies and can also be used for evaluation of tailored COVID-19 vaccines for additional booster doses in the future.

## Supplementary Data

342000 Supplementary Materials
