# Supplementary material for: Relative effectiveness of bivalent boosters against severe COVID-19 outcomes among people aged ≥ 65 years in Finland, September 2022 to August 2023
Source: Euro Surveill. 2024 Sep 12;29(37):2300587. doi: 10.2807/1560-7917.ES.2024.29.37.2300587 (PMC11395282; doi:10.2807/1560-7917.ES.2024.29.37.2300587)
Supplement: Supplementary Materials [file Supplementary_Materials_2300587.pdf]

Supplementary Information

Relative effectiveness of bivalent boosters against severe COVID-19 outcomes among people aged $\geq$ 65 years in Finland, September 2022 to August 2023

Supplementary Table S1: Enrollment of the study cohort 2
Supplementary Table S2: Severely immunocompromising conditions 3
Supplementary Table S3: Highly predisposing comorbidities or medical therapies 4
Supplementary Table S4: Moderately predisposing comorbidities or medical therapies 5
Supplementary Table S5: Hazard ratios, 65-to-110-year-olds 6
Supplementary Table S6: Hazard ratios, 65-to-110-year-olds, stratified by vaccine composition 8
Supplementary Table S7: Hazard ratios, 65-to-79-year-olds 11
Supplementary Table S8: Hazard ratios, 80-to-110-year-olds 13
Supplementary Figure S1: Distribution of sequenced Omicron lineages in Finland 15
Supplementary Figure S2: Distribution of deaths due to COVID-19 in Finland 16

This supplementary material is hosted by Eurosurveillance as supporting information alongside the article *"Relative effectiveness of bivalent boosters against severe COVID-19 outcomes among people aged $\geq$ 65 years in Finland, September 2022 to August 2023"*, on behalf of the authors, who remain responsible for the accuracy and appropriateness of the content. The same standards for ethics, copyright, attributions and permissions as for the article apply. Supplements are not edited by Eurosurveillance and the journal is not responsible for the maintenance of any links or email addresses provided therein.

Supplementary Table S1: Enrollment of the study cohort The target population included all individuals aged 65 years or more at the beginning of the study who were living in Finland during the study and had received at least two monovalent COVID-19 vaccine doses prior to the study.

	No. of individuals
Target population	1216945
Excluded due to hospitalisation due to COVID-19 at the beginning of the study	459
Excluded due to too short dosing interval* prior to the study	8616
Excluded due to bivalent booster prior to the study	27
Excluded due to right-censoring event prior to the start of individual follow-up**	15972
Study cohort	1191871

* less than 91 days between two doses or
less than 14 days between the first two doses (42 days if the first vaccination was with Vaxzevria)

** The individual follow-up period started earliest on 1 September 2022 and at least 90 days after the last monovalent COVID-19 vaccination or laboratory-confirmed SARS-CoV-2 infection prior to the study. If there was another COVID-19 vaccination or laboratory-confirmed SARS-CoV-2 infection or the individual died after the start of the study but within those 90 days, the individual was excluded.

Supplementary Table S2: Severely immunocompromising conditions Definition of comorbidities that were considered severely immunocompromising conditions.

Classification system	Codes
**Actively treated cancer**	
ICD10	C00, C01, C02, C03, C04, C05, C06, C07, C08, C09, C10, C11, C12, C13, C14, C15, C16, C17, C18, C19, C20, C21, C22, C23, C24, C25, C26, C27, C28, C29, C30, C31, C32, C33, C34, C35, C36, C37, C38, C39, C40, C41, C42, C43, C45, C46, C47, C48, C49, C50, C51, C52, C53, C54, C55, C56, C57, C58, C59, C60, C61, C62, C63, C64, C65, C66, C67, C68, C69, C70, C71, C72, C73, C74, C75, C76, C77, C78, C79, C80, C81, C82, C83, C84, C85, C86, C87, C88, C89, C90, C91, C92, C93, C94, C95, C96, C97, D051, D39
**Organ or stem cell transplantation**	
ICD10	T86, Z94
**Severe disorder of the immune system**	
ICD10	D7081, D7089, D80, D81, D82, D83, D84, E3100, E723, E76, E791, E792, E798, E799, Q7782, Q7800, Q7808, Q809, Q8900, Q8933, Q8934, Q938, Z908
NCSP	JMA

ICD10, International Statistical Classification of Diseases, tenth revision;
NCSP, Nordic Classification of Surgical Procedures

Supplementary Table S3: Highly predisposing comorbidities or medical therapies Definition of comorbidities and medical therapies that were considered highly predisposing to severe COVID-19.

Classification system	Codes
**Down syndrome**	
ICD10	Q90
**Severe chronic respiratory disease**	
ICD10	E84, J41, J42, J43, J44, J45, J46, J47, Z902
ICPC2	R96
**Severe kidney disease**	
ICD10	E102, E112, E142, I12, I13, N00, N01, N02, N03, N04, N05, N07, N08, N11, N14, N18, N19
**Type 2 diabetes mellitus**	
ATC	A10B
ICD10	E11, E13, E14
ICPC2	T90

ATC, Anatomical Therapeutic Chemical Classification System;

ICD10, International Statistical Classification of Diseases, tenth revision;

ICPC2, International Classification of Primary Care, second edition

Supplementary Table S4: Moderately predisposing comorbidities or medical therapies Definition of comorbidities and medical therapies that were considered moderately predisposing to severe COVID-19.

Classification system	Codes
**Autoimmune disease**	
ICD10	D86, K50, K51, L40, M02, M05, M06, M07, M139, M45, M460, M461, M469, M941
**Immunosuppressive medication**	
ATC	H02AB02, H02AB04, H02AB06, H02AB07, L01BA01, L01XC02, L04AA06, L04AA10, L04AA13, L04AA18, L04AA24, L04AA26, L04AA29, L04AA33, L04AA37, L04AB, L04AC, L04AD01, L04AD02, L04AX01, L04AX03
**Neurological condition affecting breathing**	
ICD10	G20, G21, G22, G23, G24, G25, G26, G70, G71, G72, G73, G80, G81, G82, G83, I60, I61, I62, I63, I64, I65, I66, I67, I68, I69
**Psychotic disease**	
ATC	N05AH02
ICD10	F20, F21, F22, F23, F24, F25, F26, F27, F28, F29
ICPC2	P72
**Severe chronic liver disease**	
ICD10	K702, K703, K704, K71, K72, K73, K74
**Severe heart disease**	
ICD10	I110, I119, I12, I130, I131, I132, I139, I15, I20, I21, I22, I23, I24, I25, I26, I27, I28, I418, I42, I43, I50
**Sleep apnea**	
ICD10	G473
NCSP	WX723, WX780
**Type 1 diabetes mellitus or adrenal insufficiency**	
ATC	A10A
ICD10	E10, E250, E271, E272, E274, E3100, E3101, E3108, E896
ICPC2	T89

ATC, Anatomical Therapeutic Chemical Classification System;

ICD10, International Statistical Classification of Diseases, tenth revision;

ICPC2, International Classification of Primary Care, second edition;

NCSP, Nordic Classification of Surgical Procedures

Supplementary Table S5: Hazard ratios, 65-to-110-year-olds Covariate-adjusted hazard ratios comparing the hazards of severe COVID-19 outcomes and a negative control outcome in 65-to-110-year-olds who received a bivalent booster with the corresponding hazards in those who did not receive a bivalent booster.

			Hazard ratio		
Days since bivalent booster	Cases	Person-years	MLE	LCI	UCI
September 2022 – February 2023					
**Hospitalisation due to COVID-19**					
No bivalent booster	1599	298530			
0–2	5	5287	0.15	0.06	0.36
3–8	37	10556			
9–13	27	8771			
14–60	186	79477	0.45	0.37	0.55
61–120	91	67702	0.70	0.54	0.92
121–180	6	3109	1.41	0.56	3.55
**Death due to COVID-19**					
No bivalent booster	974	298767			
0–2	0	5287	NE	NE	NE
3–8	1–4	10557			
9–13	11	8772			
14–60	109	79499	0.49	0.38	0.62
61–120	58	67725	0.71	0.52	0.98
121–180	1–4	3110	0.41	0.06	3.05
**Death in which COVID-19 was a contributing factor**					
No bivalent booster	812	298767			
0–2	1–4	5287	0.13	0.03	0.52
3–8	1–4	10557			
9–13	4	8772			
14–60	99	79499	0.40	0.31	0.51
61–120	57	67725	0.56	0.41	0.78
121–180	2	3110	0.71	0.17	2.98
**Emergency room visit due to injury**					
No bivalent booster	10078	296697			
0–2	100	5257	0.59	0.48	0.72
3–8	329	10493			
9–13	259	8715			
14–60	2434	78846	0.92	0.87	0.98
61–120	2105	66981	0.92	0.86	0.98
121–180	101	3072	1.08	0.87	1.32
March – August 2023					
**Hospitalisation due to COVID-19**					
No bivalent booster	468	208496			
0–2	0	67	NE	NE	NE
3–8	0	151			
9–13	0	149			
14–60	6–9	4098	0.67	0.33	1.36
61–120	61	37642	0.53	0.39	0.71
121–180	391	100232	0.88	0.74	1.05
181–240	210	98988	0.89	0.73	1.10
241–300	54	69818	1.03	0.70	1.50

(Supplementary Table S5 continued)

Days since bivalent booster	Cases	Person-years	MLE	LCI	UCI
301–364	1–4	4252	0.77	0.18	3.26
**Death due to COVID-19**					
No bivalent booster	170	208570			
0–2	0	67	NE	NE	NE
3–8	0	151			
9–13	0	149			
14–60	1–4	4099	0.70	0.22	2.22
61–120	17	37649	0.41	0.24	0.70
121–180	136	100270	0.80	0.60	1.06
181–240	106	99038	0.90	0.66	1.22
241–300	33	69833	1.28	0.77	2.13
301–364	1–4	4253	1.46	0.41	5.18
**Death in which COVID-19 was a contributing factor**					
No bivalent booster	123	208570			
0–2	0	67	NE	NE	NE
3–8	0	151			
9–13	0	149			
14–60	1–4	4099	1.16	0.36	3.72
61–120	9	37649	0.38	0.18	0.78
121–180	108	100270	1.02	0.73	1.41
181–240	66	99038	0.75	0.52	1.08
241–300	23	69833	1.20	0.67	2.14
301–364	1–4	4253	2.00	0.25	16.05
**Emergency room visit due to injury**					
No bivalent booster	6060	204335			
0–2	1–4	66	0.47	0.07	3.37
3–8	5	149			
9–13	1–4	146			
14–60	114	4031	0.94	0.78	1.13
61–120	1219	37042	1.03	0.96	1.11
121–180	3074	98513	0.96	0.91	1.02
181–240	2950	96917	0.96	0.91	1.02
241–300	1990	68141	0.96	0.90	1.03
301–364	118	4144	1.00	0.82	1.21

MLE, maximum likelihood estimate;

LCI/UCI, lower/upper limit of the 95% Wald confidence interval;

NE, not estimated

Case counts between 1 and 4 are reported as 1–4 and if necessary (due to the presentation of totals in the main text) also bigger counts are masked to not disclose any count between 1 and 4.

Supplementary Table S6: Hazard ratios, 65-to-110-year-olds, stratified by vaccine composition Covariate-adjusted hazard ratios comparing the hazards of severe COVID-19 outcomes in 65-to-110-year-olds who received a bivalent booster with the corresponding hazards in those who did not receive a bivalent booster distinguishing between BA.1 and BA.4-5 bivalent COVID-19 vaccines.

			Hazard ratio		
Days since bivalent booster	Cases	Person-years	MLE	LCI	UCI
September 2022 – February 2023					
**Hospitalisation due to COVID-19**					
No bivalent booster	1599	299128			
BA.1 bivalent booster					
0–13	19	7516	0.37	0.23	0.60
14–60	53	24634	0.38	0.28	0.52
61–120	36	21732	0.78	0.54	1.12
121–180	1–4	1649	1.99	0.72	5.49
BA.4-5 bivalent booster					
0–13	50	17109	0.49	0.36	0.67
14–60	132	54876	0.50	0.40	0.62
61–120	52	45973	0.68	0.49	0.94
121–180	1–4	1441	0.66	0.09	4.83
**Death due to COVID-19**					
No bivalent booster	973	299365			
BA.1 bivalent booster					
0–13	ND	7517	0.17	0.06	0.46
14–60	26	24640	0.38	0.25	0.57
61–120	24	21739	0.94	0.60	1.46
121–180	0	1650	NE	NE	NE
BA.4-5 bivalent booster					
0–13	ND	17110	0.20	0.11	0.37
14–60	83	54891	0.55	0.42	0.71
61–120	34	45988	0.62	0.42	0.92
121–180	ND	1442	0.76	0.10	5.72
**Death in which COVID-19 was a contributing factor**					
No bivalent booster	814	299365			
BA.1 bivalent booster					
0–13	ND	7517	0.23	0.09	0.56
14–60	30	24640	0.42	0.29	0.63
61–120	20	21739	0.68	0.42	1.10
121–180	ND	1650	1.65	0.40	6.84
BA.4-5 bivalent booster					
0–13	ND	17110	0.06	0.02	0.17
14–60	68	54891	0.40	0.30	0.53
61–120	36	45988	0.53	0.36	0.77
121–180	0	1442	NE	NE	NE
March – August 2023					
**Hospitalisation due to COVID-19**					
No bivalent booster	467	209017			
BA.1 bivalent booster					
0–13	0	32	NE	NE	NE
14–60	ND	622	0.51	0.07	3.61

(Supplementary Table S6 continued)

Days since bivalent booster	Cases	Person-years	MLE	LCI	UCI
61–120	12	10116	0.37	0.20	0.66
121–180	122	29684	0.84	0.67	1.06
181–240	78	30496	0.95	0.72	1.24
241–300	21	22346	1.14	0.70	1.87
BA.4-5 bivalent booster					
0–13	0	336	NE	NE	NE
14–60	ND	3484	0.70	0.33	1.48
61–120	48	27576	0.59	0.43	0.82
121–180	269	70618	0.93	0.77	1.12
181–240	133	68538	0.90	0.71	1.13
241–300	31	47483	0.99	0.63	1.55
BA.1 and BA.4-5 bivalent boosters					
301–364	ND	4233	0.78	0.18	3.32
**Death due to COVID-19**					
No bivalent booster	170	209091			
BA.1 bivalent booster					
0–13	0	32	NE	NE	NE
14–60	0	622	NE	NE	NE
61–120	7	10117	0.63	0.28	1.39
121–180	42	29695	0.82	0.56	1.20
181–240	42	30514	1.16	0.79	1.70
241–300	14	22351	1.64	0.87	3.08
BA.4-5 bivalent booster					
0–13	0	336	NE	NE	NE
14–60	ND	3485	0.81	0.25	2.56
61–120	10	27582	0.34	0.17	0.66
121–180	95	70645	0.82	0.60	1.10
181–240	63	68571	0.80	0.57	1.13
241–300	19	47492	1.11	0.62	2.02
BA.1 and BA.4-5 bivalent boosters					
301–364	ND	4234	1.44	0.41	5.10
**Death in which COVID-19 was a contributing factor**					
No bivalent booster	122	209091			
BA.1 bivalent booster					
0–13	0	32	NE	NE	NE
14–60	0	622	NE	NE	NE
61–120	1–4	10117	0.30	0.07	1.26
121–180	24	29695	0.74	0.46	1.21
181–240	25	30514	0.92	0.57	1.49
241–300	7	22351	1.10	0.48	2.55
BA.4-5 bivalent booster					
0–13	0	336	NE	NE	NE
14–60	ND	3485	1.33	0.41	4.24
61–120	5–8	27582	0.41	0.18	0.91
121–180	82	70645	1.17	0.82	1.65
181–240	40	68571	0.70	0.46	1.06
241–300	16	47492	1.32	0.69	2.52
BA.1 and BA.4-5 bivalent boosters					
301–364	ND	4234	2.07	0.26	16.59

(Supplementary Table S6 continued)

Days since bivalent booster	Cases	Person-years	MLE	LCI	UCI

MLE, maximum likelihood estimate;
LCI/UCI, lower/upper limit of the 95% Wald confidence interval;
ND, not disclosed;
NE, not estimated

Case counts between 1 and 4 are reported as 1–4. If a count in Supplementary Table S5 is masked, the corresponding counts in the present table (Supplementary Table S6) are not disclosed.

Supplementary Table S7: Hazard ratios, 65-to-79-year-olds Covariate-adjusted hazard ratios comparing the hazards of severe COVID-19 outcomes in 65-to-79-year-olds who received a bivalent booster with the corresponding hazards in those who did not receive a bivalent booster.

			Hazard ratio		
Days since bivalent booster	Cases	Person-years	MLE	LCI	UCI
September 2022 – February 2023					
**Hospitalisation due to COVID-19**					
No bivalent booster	696	212717			
0–13	24	17653	0.40	0.26	0.62
14–60	105	56866	0.64	0.49	0.83
61–120	48	47684	0.83	0.57	1.20
121–180	1–4	2182	1.48	0.45	4.85
**Death due to COVID-19**					
No bivalent booster	217	212826			
0–13	ND	17654	0.36	0.16	0.80
14–60	24	56877	0.46	0.28	0.75
61–120	10	47697	0.48	0.23	0.99
121–180	0	2183	NE	NE	NE
**Death in which COVID-19 was a contributing factor**					
No bivalent booster	208	212826			
0–13	ND	17654	0.11	0.03	0.47
14–60	24	56877	0.44	0.27	0.74
61–120	12	47697	0.47	0.24	0.93
121–180	ND	2183	1.07	0.14	8.38
March – August 2023					
**Hospitalisation due to COVID-19**					
No bivalent booster	267	165026			
0–13	0	286	NE	NE	NE
14–60	ND	3254	0.59	0.22	1.61
61–120	30	28461	0.51	0.33	0.76
121–180	204	72878	0.97	0.76	1.23
181–240	114	72005	0.95	0.72	1.26
241–300	27	50041	0.96	0.58	1.59
301–364	ND	3020	1.44	0.33	6.33
**Death due to COVID-19**					
No bivalent booster	57	165068			
0–13	0	286	NE	NE	NE
14–60	ND	3254	0.75	0.10	5.58
61–120	1–4	28466	0.35	0.12	1.02
121–180	39	72897	0.87	0.52	1.44
181–240	27	72033	0.80	0.46	1.41
241–300	13	50049	2.00	0.87	4.61
301–364	ND	3021	1.96	0.22	17.53
**Death in which COVID-19 was a contributing factor**					
No bivalent booster	45	165068			
0–13	0	286	NE	NE	NE
14–60	ND	3254	1.95	0.45	8.42
61–120	1–4	28466	0.35	0.10	1.20
121–180	37	72897	1.22	0.70	2.13
181–240	16	72033	0.65	0.33	1.28

(Supplementary Table S7 continued)

Days since bivalent booster	Cases	Person-years	MLE	LCI	UCI
241–300	5	50049	0.80	0.26	2.47
301–364	0	3021	NE	NE	NE

MLE, maximum likelihood estimate;

LCI/UCI, lower/upper limit of the 95% Wald confidence interval;

ND, not disclosed;

NE, not estimated

Case counts between 1 and 4 are reported as 1–4. If a count in Supplementary Table S5 is masked, the corresponding counts in the present table (Supplementary Table S7) are not disclosed.

Supplementary Table S8: Hazard ratios, 80-to-110-year-olds Covariate-adjusted hazard ratios comparing the hazards of severe COVID-19 outcomes in 80-to-110-year-olds who received a bivalent booster with the corresponding hazards in those who did not receive a bivalent booster.

			Hazard ratio		
Days since bivalent booster	Cases	Person-years	MLE	LCI	UCI
September 2022 – February 2023					
**Hospitalisation due to COVID-19**					
No bivalent booster	903	86411			
0–13	45	6972	0.47	0.33	0.67
14–60	80	22644	0.33	0.24	0.43
61–120	40	20020	0.63	0.43	0.94
121–180	1–4	908	1.47	0.34	6.37
**Death due to COVID-19**					
No bivalent booster	756	86539			
0–13	ND	6973	0.14	0.07	0.28
14–60	85	22655	0.50	0.38	0.66
61–120	48	20030	0.80	0.56	1.14
121–180	ND	909	0.57	0.08	4.30
**Death in which COVID-19 was a contributing factor**					
No bivalent booster	606	86539			
0–13	ND	6973	0.10	0.05	0.23
14–60	74	22655	0.38	0.29	0.51
61–120	44	20030	0.60	0.42	0.87
121–180	ND	909	0.57	0.07	4.28
March – August 2023					
**Hospitalisation due to COVID-19**					
No bivalent booster	200	43992			
0–13	0	82	NE	NE	NE
14–60	ND	853	0.76	0.28	2.07
61–120	30	9231	0.56	0.37	0.86
121–180	187	27425	0.83	0.64	1.07
181–240	97	27029	0.88	0.65	1.21
241–300	25	19787	1.26	0.70	2.26
301–364	0	1213	NE	NE	NE
**Death due to COVID-19**					
No bivalent booster	113	44023			
0–13	0	82	NE	NE	NE
14–60	ND	853	0.68	0.17	2.81
61–120	13	9234	0.44	0.24	0.83
121–180	98	27442	0.79	0.56	1.11
181–240	78	27052	0.97	0.67	1.39
241–300	20	19794	1.03	0.55	1.95
301–364	ND	1214	1.28	0.27	5.99
**Death in which COVID-19 was a contributing factor**					
No bivalent booster	77	44023			
0–13	0	82	NE	NE	NE
14–60	ND	853	0.62	0.09	4.57
61–120	6	9234	0.41	0.17	1.00
121–180	69	27442	0.95	0.63	1.43
181–240	49	27052	0.81	0.52	1.26

(Supplementary Table S8 continued)

Days since bivalent booster	Cases	Person-years	MLE	LCI	UCI
241–300	18	19794	1.43	0.72	2.84
301–364	ND	1214	3.62	0.41	31.93

MLE, maximum likelihood estimate;

LCI/UCI, lower/upper limit of the 95% Wald confidence interval;

ND, not disclosed;

NE, not estimated

Case counts between 1 and 4 are reported as 1–4. If a count in Supplementary Table S5 is masked, the corresponding counts in the present table (Supplementary Table S8) are not disclosed.

Supplementary Figure S1: Distribution of sequenced Omicron lineages in Finland The graph shows the percentages of the most frequent SARS-CoV-2 sublineages detected by sentinel surveillance and recorded in the National Infectious Diseases Register by week of sample collection since end of September 2022. Green bars represent BA.2 sublineages (BA.2 lineages and CH.1.1.X), orange bars represent BA.4 sublineages and pink bars represent BA.5 sublineages (BA.5 lineages and BQ.1.X). The greyscale bars represent recombinant lineages (XBB lineages and EG.5.X) as well as the group 'Other variants', which comprises all sublineages not assigned to any of the previous groups. The .X extension covers the whole sublineage family, e.g. BQ.1.X covers all descendants of BQ.1, such as BQ.1.1 and BQ.1.2.

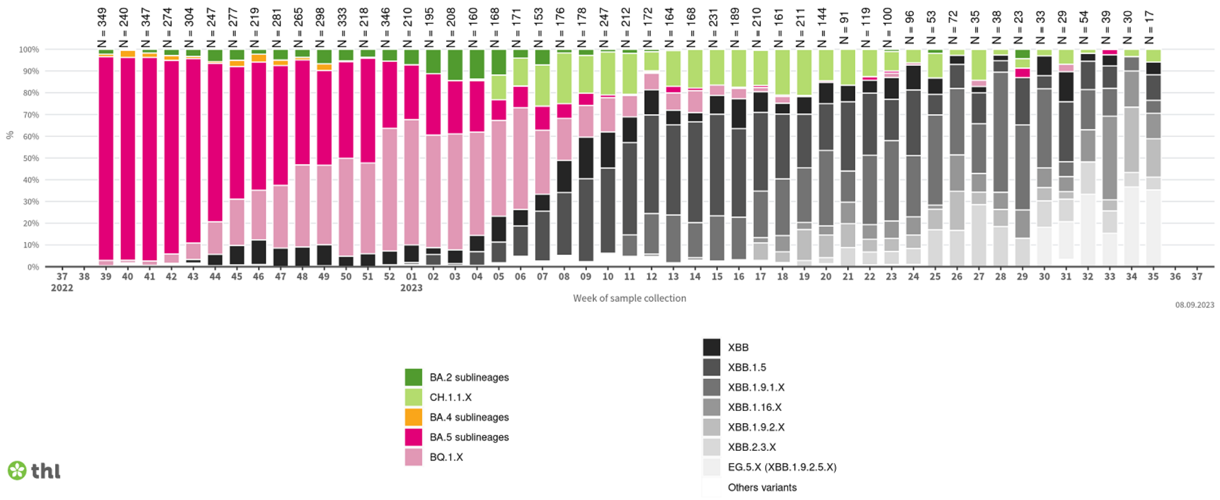

Adapted from https://thl.fi/en/web/infectious-diseases-and-vaccinations/what-s-new/coronavirus-covid-19-latest-updates/coronavirus-variants/genomic-surveillance-of-sars-cov-2

Supplementary Figure S2: Distribution of deaths due to COVID-19 in Finland The graph shows the weekly number of deaths due to COVID-19 and deaths in which COVID-19 was a contributing factor in Finland.

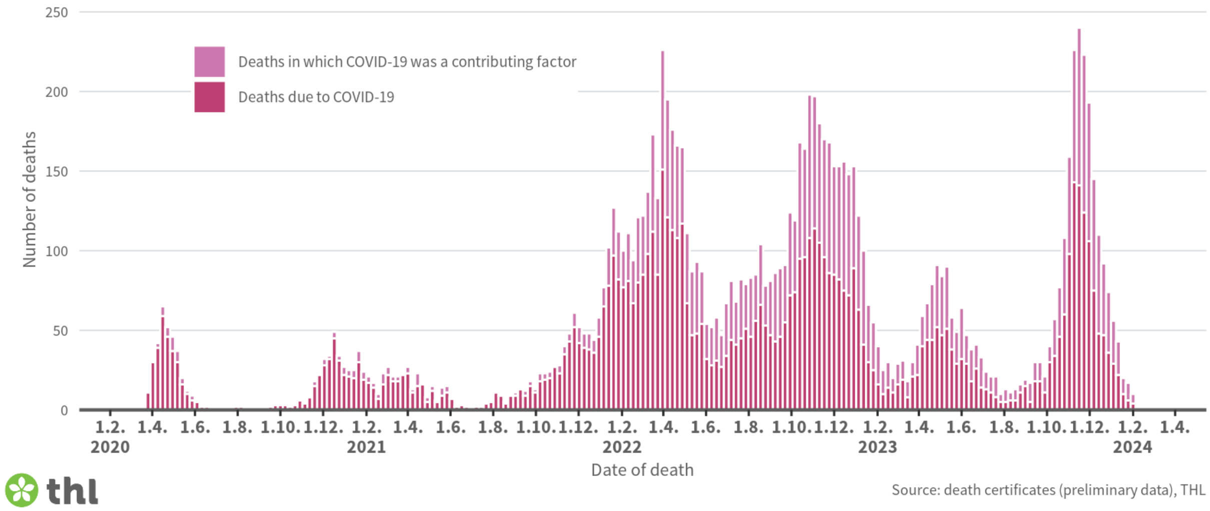

Adapted from https://www.thl.fi/episeuranta/tautitapaukset/coronamap.html
